# Hypertrophy of the Tonsils

**Published:** 1891-01

**Authors:** T. Hilliard Wood

**Affiliations:** Nashville, Tenn.


### Southern Medical Record.

A MONHLY JOURNAL OF MEDICINE AND SURGERY.

VOL. XXI. ATLANTA, GA., JANUARY, 1891. No. 1.

### Original Articles.

### HYPERTROPHY OF THE TONSILS.

BY T. HILLIARD WOOD, M. D., NASHVILLE, TENN.*

Hypertrophy of the tonsils is a disease almost as old as the history of medicine, and has occurred with sufficient frequency to claim at all times the serious consideration of surgeons. But the treatment employed for its relief has been subject to many variations.

The mucous membrane covering the tonsils presents the openings of from twelve to fifteen follicles, whose function it it is to secrete a fluid to lubricate the bolus of food in deglutition. Those follicles penetrate into the substance of the tonsil, and terminate by blind extremeties. Filling the space between these follicles and surrounding their caecal extremeties is a quantitity of adenoid tissue. In this adenoid tissue we have as a rule the beginning of tonsillar hypertrophy.

The causes giving rise to hypertrophy of the tonsils may be divided into two classes; predisposing and exciting.

Of the predisposing causes, few are so potent as age. Hypertrophy of the tonsils is essentially a disease of children and young adults. Although seldom congenital, it is frequently observed in the first years of life, but more often occurs between the fifth and twentieth years. Not more than one case

*Read at the meeting of the Tri-State Medical Association of Alabama, Georgia and Tennessee, October 15th, 1890, Chattanooga, Tenn.

in ten will be over thirty years of age, and beyond the fortieth year it is rarely observed.

All writers agree that among the predisposing causes a cachectic state of the system, particularly that due to struma is prominent, sex must also be considered, as hypertrophy occurs about twice as often in the male as in the female.

Among the exciting causes may be mentioned acute tonsillitis, pharyngitis, obstruction of the posterior nares as by a polypus. Or syphilis, either hereditary or acquired, may produce chronic inflammation and enlargement of the tonsils. The enlargement may have its origin in the throat affection accompanying diphtheria, scarlatina, measles or smallpox. So may difficult dentition, as in cutting a wisdom tooth, cause it.

The enlargement may be at once seen if by depressing the tongue we look into the fauces. The degree of enlargement varies in different cases from the slightest possible increase to the size of a guinea's egg, when they almost meet in the median line. The enlarged tonsil produces a feeling as if a foreign body were in the throat, and consequently a desire to swallow. The hypertrophy, though generally affecting both tonsils, usually causes a greater enlargement of the one than the other. The effect of the enlargement on the features is well illustrated by Mackensie, who says, "We often predicate the existence of enlarged tonsils as the child with open mouth, drooping eyelids, dull expression and thick voice enters the consultation room.

Slight enlargement of the tonsils seems compatible with good health, as the affection often is not noticed by the patient, until the enlargement interferes mechanically with the physiological functions of the pharynx. The enlargement causes a narrowing of the pharynx with difficult inspiration and expiration, the inspiration being the more embarrassed. This obstruction to inspiration causes an extra exertion on the part of the diaphragm, and this constant and increased traction of the diaphragm on the thoracic walls causes a narrowing or constriction of the chest corresponding to the attachment of the diaphragm. This "pigeon breast" is best developed where the tonsillar enlargement occurs in young patients in whom the osseo-cartilaginous frame work of the thorax is soft and yielding.

The enlarged tonsil by filling up the pharynx and obstructing free nasal respiration causes the patient to breathe with mouth open, both when awake and during sleep. The air blast through the nose being thus interfered with, the secretions are not dried up and driven from the nose as in health, but remain to decompose and set up inveterate cases of nasal catarrh.

Again the enlarged tonsil is a frequent cause of impaired hearing. This result is brought about, according to some, by the tonsils pressing on the opening of the eustachian tube and so intefering with the ventilation of the middle ear. According to others, another explanation, and the one more probable is that the weight and traction of the tonsil produce a flatulency of the eustachian tube, and the inflammation of the nasal mucous membrane extending up this tube causes the deafness. The movements of the soft palate and pharynx being interfered with, the voice is altered in tone, becoming thick and gutteral. Sleeping with mouth open and snoring, and hence on awakening find the mouth and pharynx dry, are frequent symptoms of enlarged tonsils.

The obstruction to deglutition, though less harmful than the obstruction to respiration, has nevertheless a baneful influence by interfering with the proper nutrition of the patient, and thus the patient, if a child, grows up in a state of chronic bad health caused by a diminished supply of oxygen and food.

Perversion of the sense of smell, due most likely to the accompanying catarrh, not infrequently occurs with hypertrophied tonsils. Frequent attacks of acute tonsillitis upon slight exposure to cold are often observed.

Most cases of enlarged tonsils have their origin in some acute inflammation of these glands. The adenoid tissue of the tonsil is likely causative in maintaining this hyperaemia until the stage of true hypertrophy is developed.

Enlargement of the tonsils is a true hypertrophy, an increase in the amount of each constituent element. The epithelial layer over the tonsil is thickened, and the mucosa is itself infiltrated with cells and new tissue elements. The glands are increased in size and have a multiplication of their tissues and follicles. But the great increase in the size of the tonsil is due to the development of connective tissue interspersed with bands of fibrous tissue.

Enlarged tonsils do not of themselves directly threaten life, although some isolated cases of death from suffocation are on record. If left to themselves the new tissue they contain will, in time, undergo contraction and by the age of thirty or shortly after the enlarged tonsil will have returned to a minimum size.

But although all cases tend to spontaneous cure, if left to themselves they produce discomfort, retard the growth and development of the child, and during inflammatory affections of the air passages, become directly dangerous to life. For these reasons they should receive prompt and efficient treatment.

The treatment for the reduction of enlarged tonsils is divided by Mackensie into local, constitutional and operative.

First, as to local treatment: If the enlargement be due to swelling of the mucous membrane, or to engorgement and congestion of the tonsil, the application of astringents may be of service. The most useful local remedies are the sub-sulphate and per-chloride of iron, about one to six or eight in water or glycerine, and alum or tannin in powder.

But where there is real overgrowth, the remedy, as Mackensie well observes, must be of a destructive character, and escharotics, not astringents, must be used. Among escharotics, London paste is useful and should be applied once or twice a week. This will produce a slough, and repeated applications will reduce the gland to the normal size. The treatment by escharotics, while effectual in the end, is quite tedious and painful. When for any reason excision cannot be performed, Donaldson, of Baltimore, incises the tonsil, inserting into each incision a crystal of chromic acid.

Constitutional measures to effect the reduction of the gland includes remedies to combat the diathesis upon which the enlargement often depends, such as the iodide of potassium, and cod liver oil and the general tonics, as the preparations of iron and the bitter tonics.

Operative treatment: Although the writer has used most of the foregoing remedies in numbers of cases, he has yet to derive from them any decided reduction of the enlargement. The reduction of the enlargement can usually only be brought about by the use of some of the operations devised for that purpose. The operation of excision of the tonsils is a very old one, having been clearly described by Celsus during the first century. From that time the popularity of the operation fluctuated, being at times highly recommended and at other times condemned. The arguments against it were, the difficulty of its performance and the danger of hemorrhage. In 1757, however, Caque proved the dread of hemorrhage had been much overrated and that the resulting wound healed readily.

From that time the operation has grown in favor and the methods of its performance have been numerous; almost every operator varying the procedure to suit his individual taste.

Of all the operations, excision by the tonsillotome is most popular, though many persons, among them the writer, prefer the bistoury and vulsellum forceps. This operation is rendered painless by applying to the tonsil a solution of cocaine and injecting, with a hypodermic syringe, a few drops of the same solution into the substance of the gland. The cocaine will also have a happy effect in controlling subsequent hemorrhage. General anesthetics, as a rule, should not be used.

The only danger which can attach to the operation is the very slight one of hemorrhage. This has been held up by conservative men as sufficient to condemn the operation. Now let us examine into the matter of hemorrhage after tonsillotomy.

Sajous says: "Profuse hemorrhage occurs in perhaps one out of every five hundred operations, while an alarming flow does not occur in one out of a thousand." Mackensie, during a long experience, never lost a case from bleeding, and only remembers to have seen it once become severe. Still statistics show hemorrhage to occur in about one per cent. of tonsillotomies, and in one thousand operations collected by Solis Cohen, there were four deaths.

In the records of the Surgeon-General's office, Washington, for the past twenty-five years there are found only two deaths following tonsillotomy, and in one of these, a patient eight years old, the cause was an anomalous course of the internal carotid. The arteries supplying the tonsil are small, and experience teaches that the hemorrhage after tonsillotomy ceases spontaneously, or is easily controlled by mild means, such as nitrate of silver, ice water gargle, or cold cloths applied to the neck. Should the bleeding still continue, we may use Mackensie's tanno-gallic gargle or the per-chloride of iron, torsion, pressure directly applied, ligature of the stump or the actual cautery. Should these fail, ligature of the common carotid may be done, and in three recorded cases in which the common carotid was ligated, recovery resulted in all. So that the alarming danger of hemorrhage resolves itself down to two deaths in the past twenty-five years, and there is nothing to show that the above means to arrest bleeding were employed in these two cases. Still it may be justly urged that all the deaths after tonsillotomy were not reported, so that the entire number of fatal cases, after all, is not so small.

The well known fact that adults are much more liable to this hemorrhage than children, is well illustrated by Wright, of Brooklyn. He says: "But while the danger from hemorrhage in children in whom the tonsillar tissue is loose and lax and will therefore readily collapse and arrest bleeding, is almost nil; yet in adult tonsils the fibroid elements now in excess and which relatively increase as age advances, form a tissue less favorable for the retraction and closure of the cut vessel than the spongy mass of a young tonsil made up largely of lymphoid tissue."

In order to avoid, as far as possible, any danger of hemorrhage, we have the comparatively bloodless operations, as the cold snare, ignipuncture and the galvano-cautery amygdalotome. Of these the galvano-cautery amydalotome seems preferable. Ignipuncture is slow, requiring repeated applications attended by considerable pain. Moreover, it cannot be employed in the cases of refractory children.

